# Respiratory support strategy in adults with acute hypoxemic respiratory failure: a systematic review and network meta-analysis

**DOI:** 10.1186/s40981-022-00525-4

**Published:** 2022-05-06

**Authors:** Hiromu Okano, Masaaki Sakuraya, Tomoyuki Masuyama, Shunsuke Kimata, Satoshi Hokari

**Affiliations:** 1 Department of Critical Care and Emergency Medicine, National Hospital Organization Yokohama Medical Center, 3-60-2 Harajuku, Totsuka-ku, Yokohama-shi, Kanagawa 245-8575 Japan; 2 International University of Health and Welfare Graduate School of Public Health, 4-1-26 Akasaka, Minato City, Tokyo 107-8402 Japan; 3 Department of Emergency and Intensive Care Medicine, JA Hiroshima General Hospital, 1-3-3 Jigozen, Hatsukaichi-City, Hiroshima 738-8503 Japan; 4 Department of Emergency and Critical Care Medicine, Misato Kenwa Hospital, 4-494-1 Takano, Misato-shi, Saitama, 341-8555 Japan; 5 Department of Preventive Services, School of Public Health, Kyoto University, Yoshida-konoe-cho, Sakyo-ku, Kyoto, 606-8501 Japan; 6 Department of Respiratory Medicine and Infectious Diseases, Niigata University Graduate School of Medical and Dental Sciences, 1-757 Asahimachidori, Chuo-ku, Niigata, 951-8510 Japan

**Keywords:** Noninvasive ventilation, High-flow nasal oxygen, Acute hypoxemic respiratory failure, Network meta-analysis

## Abstract

**Introduction:**

Network meta-analyses (NMAs) of respiratory management strategies for acute hypoxemic respiratory failure (AHRF) have been reported, but no previous study has compared noninvasive ventilation (NIV), high-flow nasal oxygen (HFNO), standard oxygenation therapy (SOT), and invasive mechanical ventilation (IMV) for de novo AHRF. Therefore, we conducted an NMA to assess the effectiveness of these four respiratory strategies in patients with de novo AHRF.

**Methods:**

The Cochrane Central Register of Controlled Trials, MEDLINE, EMBASE, and Ichushi databases were searched. Studies including adults aged ≥18 years with AHRF and RCTs that compared two different oxygenation techniques (SOT, NIV, HFNO, or IMV) were selected. A frequentist-based approach with multivariate random-effects meta-analysis was used. The outcomes were mortality and intubation rates.

**Results:**

Among the 14,263 records initially identified, 25 studies (3302 patients) were included. In the analysis of mortality, compared to SOT, NIV (risk ratio [RR], 0.76; 95% confidence interval [CI], 0.61–0.95) reduced mortality; however, IMV (RR, 1.01; 95% CI, 0.57–1.78) and HFNO (RR, 0.89; 95% CI, 0.66–1.20) did not. For assessments of the intubation incidence, compared to SOT, NIV use (RR, 0.63; 95% CI, 0.51–0.79) was associated with a reduction in intubation, but HFNO (RR, 0.82; 95% CI, 0.61–1.11) was not significant.

**Conclusions:**

Our NMA demonstrated that only NIV showed clinical benefits compared with SOT as an initial respiratory strategy for de novo AHRF. Further investigation, especially comparison with HFNO, is warranted.

**Trial registration:**

PROSPERO (registration number: CRD42020213948, 11/11/2020).

**Supplementary Information:**

The online version contains supplementary material available at 10.1186/s40981-022-00525-4.

## Background

Acute hypoxemic respiratory failure (AHRF) is frequently found in critically ill patients and associated with poor outcomes. Noninvasive respiratory strategies, including noninvasive ventilation (NIV) and high-flow nasal oxygen (HFNO), have been investigated as an initial respiratory support for patients with AHRF. NIV is recommended to reduce the risk of endotracheal intubation and mortality in patients with AHRF due to cardiopulmonary edema [1]. However, the efficacy of NIV has not been consistent among patients with AHRF because it can occur due to various factors. De novo AHRF is defined as significant hypoxemia in the absence of chronic lung disease and excluding respiratory failure occurring in the immediate postoperative or post-extubation period [2]. Recent clinical practice guidelines do not recommend NIV but HFNO for patients with de novo AHRF, based on the evidence compared with standard oxygen therapy (SOT) [3, 4].

Acute respiratory distress syndrome (ARDS) is a major cause of de novo AHRF. As an initial management among noninvasive and invasive respiratory strategies, NIV is recommended for patients with mild ARDS [5]. In the post hoc analysis of the LUNG SAFE study, NIV was used in ARDS patients (15%) [6]. In contrast, HFNO is preferred to manage de novo AHRF patients with novel coronavirus disease 2019 (COVID-19), compared with SOT and NIV [7]. HFNO has also gained attention as an initial respiratory management, compared with early initiation of invasive mechanical ventilation (IMV) [8]. Thus, noninvasive respiratory strategies are commonly used to manage patients with de novo AHRF, despite inconclusive evidence.

Although noninvasive respiratory strategies are expected to improve clinical outcomes by avoiding endotracheal intubation and its adverse events [9], treatment failure and delayed intubation contribute to poor outcomes [6, 10–12]. No meta-analyses have been reported to compare noninvasive respiratory strategies with IMV in patients with AHRF. It is warranted to clarify which noninvasive respiratory strategy is effective in avoiding endotracheal intubation and to evaluate the efficacy in reducing mortality for patients with de novo AHRF as an initial respiratory strategy, compared with SOT and IMV. Therefore, we conducted a network meta-analysis (NMA) to compare the four respiratory strategies (NIV, HFNO, SOT, and IMV) as an initial strategy in adult patients with de novo AHRF.

### Research question

Which is the most effective respiratory strategy among NIV, HFNO, SOT, and IMV in patients with de novo AHRF?

## Methods

### Protocol and registration

This systematic review was conducted in accordance with the methods recommended in the Preferred Reporting Items for Systematic Reviews and Meta-Analyses (PRISMA) 2015 guidelines [13]. The protocol has been registered in PROSPERO, a prospective international register of systematic reviews of the National Institute for Health Research and Center for Reviews and Dissemination at the University of York (http: //www.crd.york.ac.uk/PROSPERO/; registration no. CRD42020213948, on 11/11/2020).

### Inclusion criteria

#### Types of studies

We included randomized controlled trials (RCTs) that compared two of the following four methods: SOT (low-flow nasal cannula, facemask, and venturi mask with limitless flow rate) and NIV (mask type, ventilation duration, management during the interval, and methods of weaning were not limited). Additionally, studies were selected regardless of the mode, i.e., continuous positive airway pressure or pressure support ventilation, HFNO (the flow rate and fraction of inspired oxygen were not limited), and IMV (mechanical ventilation via endotracheal intubation, not tracheostomy).

#### Types of outcomes

The outcome measures included a primary outcome of short-term mortality at the end of the follow-up period (≤ 100 days). The secondary outcomes included incidence of intubation during intensive care unit (ICU) stay, ventilator-free days, and adverse events reported as any critical events by the authors of each study.

#### Types of patients

We included patients aged >18 years who had acute respiratory failure defined by new-onset (<7 days) of clinical signs (e.g., tachypnea, increased work of breathing), radiologic signs (unilateral or bilateral chest radiograph opacities), and hypoxemia. Hypoxemia was defined as the ratio of arterial oxygen partial pressure to fractional inspired oxygen (P/F ratio) below 300, SaO_2_/SpO_2_ <94% at and PaO_2_ <60 mmHg at room air or <80 mmHg with O_2_. We considered studies that included patients treated in the ICU, intermediate care unit, and emergency department.

### Exclusion criteria

#### Types of studies

Randomized crossover trials, cluster-randomized trials, and quasi-experimental trials were excluded.

#### Types of patients

We excluded patients who met the following criteria: hypercapnia (PaCO_2_ >50 mmHg), with congestive heart failure, chronic obstructive pulmonary disease (COPD), or asthma as the cause of respiratory failure, post-extubation respiratory failure, post-surgical, and post-trauma constituting >50% of the study population; had not provided informed consent and had provided do-not-resuscitate orders; and had undergone interventions limited to the emergency department or pre-hospital care.

### Search strategy

Databases used for the search were PubMed (Supplemental e-Table 1 a), Cochrane Central Register of Controlled Trials (CENTRAL; Supplemental e-Table 1 b), EMBASE (Supplemental e-Table 1 c), and Ichushi, a database of Japanese papers (Supplemental e-Table 1 d). The languages in which the studies were conducted were restricted to English and Japanese. A literature search was performed from the database inception up to June 22, 2020. A literature search was also performed from the inception of the database up to May 30, 2021. This systematic review was conducted for clinical practice guidelines for the ARDS management in Japan, and we included articles in English and Japanese only.

### Selection of the studies and data extraction

At the first screening, two of the three physicians (HO, SK, and SH) analyzed the title and abstract. At the second screening, the full text of the relevant studies was studied, and data were extracted independently from the included studies onto the standardized data-recording forms. Disagreements were resolved by discussing with one of the three physicians not involved in screening the studies. We also asked the original authors for additional details when necessary. For example, we contacted the authors if only abstracts were available, and the information was insufficient to determine whether the study met our review criteria. In cases involving discrepancies between the two reviewers, an agreement was reached through discussion or by including a third reviewer, if necessary.

We extracted the following study characteristics: methods (design, total duration, number and locations, setting, withdrawals, and date of the study); participants (number, mean age, age range, sex, the severity of the condition, diagnostic criteria, and inclusion and exclusion criteria), interventions (intervention and comparison methods), and outcomes (specified and collected primary and secondary outcomes, and time points reported).

### Quality assessment

#### Risk of bias within individual studies

The risk of bias of outcomes in the included studies was assessed independently by two of the five authors (HO, TM, SH, SK, and MS) using a modified version of the Cochrane “Risk of Bias” instrument [14]. They assessed the overall risk of bias as the worst in any of the following domains: from the randomization process, deviations from intended interventions, missing outcome data, measurement of the outcomes, and selection of the reported results. The risk of each bias was graded as “low risk of bias,” “some concerns,” or “high risk of bias.” Discrepancies between two reviewers were resolved through discussion among themselves or with a third reviewer, as necessary.

### Planned methods of analyses

#### Direct comparison meta-analysis

A pair-wise meta-analysis was performed using Review Manager (RevMan) 5.3 (RevMan 2014) [15]. Forest plots were used for the meta-analysis, and the effect size was expressed as risk ratio (RRs) with 95% confidence intervals (CIs) for the categorical data. The outcome measures were pooled using a random-effects model for the measure of study-specific effects. For all the analyses, a two-sided *P* value <0.05 was considered to be statistically significant.

#### Network comparison meta-analysis

##### Data synthesis

An NMA was performed using a frequentist approach with Confidence in Network Meta-Analysis (CINeMA) [16]. The network RR was estimated based on both direct and indirect comparisons. We constructed forest plots of the RRs with 95% CIs for each treatment strategy in the network.

##### Ranking

Ranking plots (rankograms) were constructed based on the probability that a given treatment had the highest event rate for each outcome. The surface under the cumulative ranking curve (SUCRA), which is a simple transformation of the mean rank, was used to determine the treatment hierarchy [17]. Higher values of the SUCRA statistic, which range from 0 to 100%, increase the likelihood that a therapy is ranked amongst the best in an NMA [18]. We performed ranking analysis using the mvmeta command in Stata 15.1 (StataCorp LLC, College Station, TX, USA).

### Assessment of inconsistency

Study heterogeneity among trials for each outcome was assessed by inspecting the forest plots visually and using the *I*^2^ statistic to quantify any inconsistencies [19]. Publication bias was assessed visually using a funnel plot [18]. Coherence in NMA referred to consistency in the estimates of treatment effects between direct and indirect comparisons [20]. For each pair-wise comparison, we assessed the global inconsistency test with a fitting design-by-treatment model was used to identify the disagreement between the direct and indirect estimates as a measure of inconsistency [21]. The transitivity assumption was evaluated by comparing the distribution of potential effect modifiers across treatment comparisons [22]. We used a side-splitting approach as a local method and the design-by-treatment model as a global method to detect inconsistency in the network [23]. We also estimated the prediction intervals in the results to express the impact of the common heterogeneity assumed across comparisons.

### The certainty of evidence for each network comparison

The certainty of the network estimates of the primary outcomes was assessed using the framework of CINeMA. The CINeMA approach is based on the Grades of Recommendation, Assessment, Development and Evaluation Working Group (GRADE) framework, which covers six domains of the certainty of evidence: within-study bias, across-studies bias, indirectness, imprecision, heterogeneity, and inconsistency [24].

### Sensitivity analysis

The effect of NIV varies depending on the severity of hypoxemia [5]. Sensitivity analyses, which excluded patients with mild hypoxemia (mean P/F ratio **≥**200) and type II respiratory failure, were conducted to assess the heterogeneity of clinical study participants and interventions. We also performed a sensitivity analysis; studies that reported long-term mortality (**≥**2-month mortality) were excluded.

### Difference between protocol and review

Differences between the protocol and studies included in this review were noted. In the protocol, short-term mortality was defined as within 90 days, but among the included studies, we found a study that reported 100-day mortality. Therefore, the definition of mortality was changed from within 90 to 100 days because we thought that increasing the sample size would improve the accuracy of the study. In addition, we performed a post-hoc sensitivity analysis for short-term mortality according to the follow-up period, given that a wider follow-up period range might contribute to inconsistency.

## Results

### Study selection

**Fig. 1:**
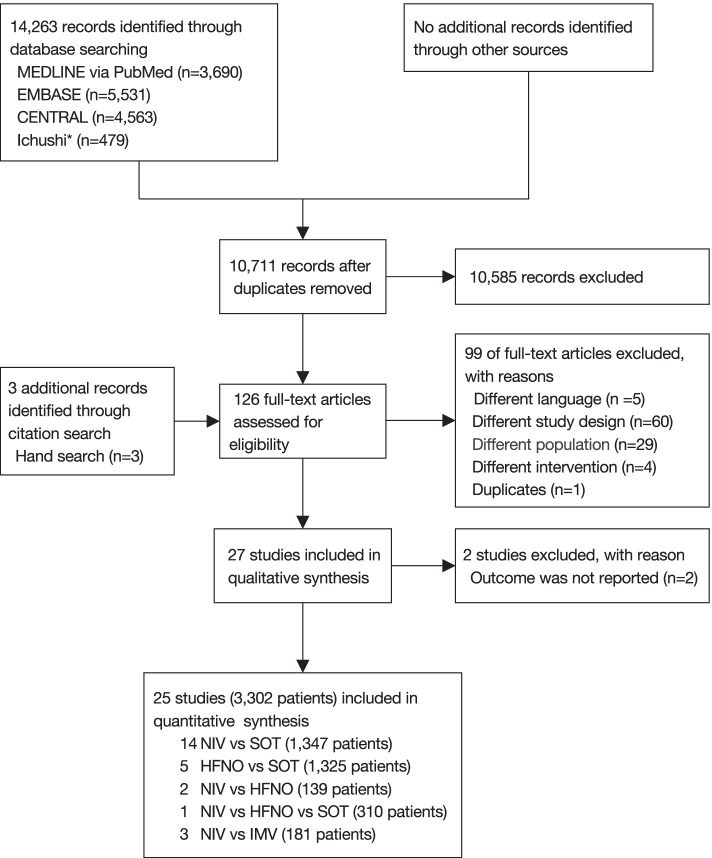
Flow diagram for the studies included in this review. ^*^Ichushi is a database of Japanese research papers. Abbreviations: CENTRAL (Cochrane Central Register of Controlled Trials); CPAP (continuous positive airway pressure); HFNO (high-flow nasal oxygen); IMV (invasive mechanical ventilation); NIV (noninvasive ventilation); RCT (randomized controlled trial); SOT (standard oxygen therapy)

**Fig. 2:**
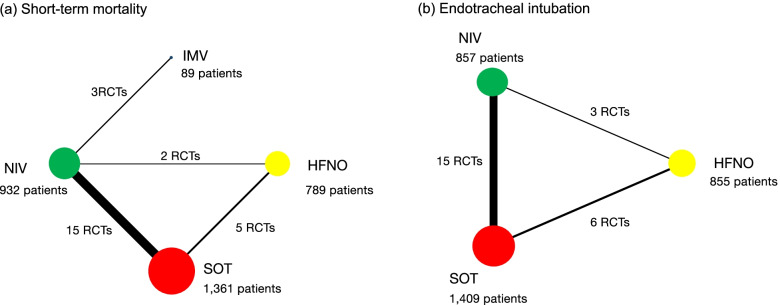
Network plot of noninvasive oxygenation strategies for adults with AHRF. When RCTs for direct comparisons were available, they were shown by connections between the nodes. The size of the node represents the number of participants who received the intervention. The thickness of the lines connecting the nodes represents the number of trials for that comparison. Abbreviations: HFNO (high-flow nasal oxygen); IMV (invasive mechanical ventilation); NIV (noninvasive ventilation); RCT (randomized controlled trial); SOT (standard oxygen therapy)

We searched 14,263 records (Fig. 1). After the study selection process, 25 studies [12, 25–48] were included in this NMA. The network structures for short-term mortality and intubation are shown in Fig. 2a, b, respectively.

### Study characteristics

The characteristics of each study included in the final dataset of the meta-analysis are summarized in Table 1. Quantitative analysis included 3302 patients. Five trials compared HFNO with SOT [25, 40, 43, 45, 46], fourteen compared NIV with SOT [27–38, 41, 44], three compared NIV with IMV [26, 42, 47], two compared NIV with HFNO [39, 48], and one compared NIV with HFNO and SOT [12].

**Table 1:** Summary of the characteristics of the studies included in the network meta-analysis

Source	Total no. of patients	Main reason for hypoxemic respiratory failure (main baseline risk factor)	Main exposure	Comparator	P/F ratio (mean)	Outcomes of interest assessed
Wysocki [27] 1995	41	Mixed AHRF (CAP 39.0%, CPE 34.1%)	NIV (*n* = 21)	SOT (*n* = 20)	207	Mortality, intubation
Antolnelli [26] 1998	64	Mixed AHRF (CPE 19%, atelectasis 25%)	NIV (*n* = 32)	IMV (*n*=32)	120	Mortality
Confalonieri [29] 1999	56	CAP	NIV (*n* = 28)	SOT (*n* = 28)	175	Mortality, intubation
Antonelli [28] 2000	40	Mixed AHRF (ARDS 37.5%, atelectasis 25%)	NIV (*n* = 20)	SOT (*n* = 20)	NA	Mortality, intubation
Delcaux [30] 2000	123	Mixed AHRF (CAP 54.5%)	NIV (*n* = 62)	SOT (*n* = 61)	144	Mortality, intubation
Martin [32] 2000	61	Mixed AHRF (COPD 38%)	NIV (*n* = 32)	SOT (*n* = 29)	199	Mortality, intubation
Hilbert [31] 2001	52	CAP in immunocompromised patients	NIV (*n* = 26)	SOT (*n* = 26)	139	Mortality, intubation
Ferrer [34] 2003	105	Mixed AHRF (CAP 32.4%, CPE 28.6%)	NIV (*n* = 51)	SOT (*n* = 54)	103	Mortality, intubation
Cosentini [33] 2010	47	CAP	NIV (*n* = 20)	SOT (*n* = 27)	248	Mortality, intubation
Squadrone [35] 2010	40	Mixed AHRF in immunocompromised patients	NIV (*n* = 20)	SOT (*n* = 20)	269	Mortality, intubation
Wermke [37] 2012	86	CAP in immunocompromised patients	NIV (*n* = 42)	SOT (*n* = 44)	270	Mortality, intubation
Zhan [38] 2012	40	ALI (immunocompromised 30%)	NIV (*n* = 21)	SOT (*n* = 19)	230	Mortality, intubation
Brambilila [36] 2014	81	CAP (immunocompromised 32%)	NIV (*n* = 40)	SOT (*n* = 41)	141	Mortality, intubation
Azevedo [39] 2015	30	CPE (43.3%), CAP (33.3%)	NIV (*n*=16)	HFNO (*n*=14)	NA	Intubation
Frat [12] 2015	310	Mixed ARF (CAP 63.5%)	NIV (*n* = 110)	HFNO (*n* = 106) SOT (*n* = 94)	155	Mortality, intubation
Lamiale [40] 2015	100	Mixed AHRF in immunocompromised patients (sepsis related 50%)	HFNO (*n* = 52)	SOT (*n* = 48)	114	Intubation
Lemiale [41] 2015	374	Pneumonia in immunocompromised patients	NIV (*n* = 191)	SOT (*n* = 183)	142	Mortality, intubation
Jones [43] 2016	303	Mixed AHRF (COPD 23.9%, Pneumonia 23.8%)	HFNO (*n* = 165)	SOT (*n* = 138)	NA	Mortality, intubation
Muncharaz [42] 2017	65	Mixed AHRF (CAP 63.1%)	NIV (*n* = 34)	IMV (*n* = 31)	97	Mortality
Azoulay [25] 2018	776	Mixed AHRF in immunocompromised patients (Pneumonia 53.0%)	HFNO (*n* = 388)	SOT (*n* = 388)	132	Mortality, intubation
He [44] 2019	200	CAP	NIV (*n* = 102)	SOT (*n* = 98)	231	Mortality, intubation
Andino [45] 2020	46	Mixed AHRF (CAP 30%)	HFNO (*n* = 24)	SOT(*n*=22)	96	Mortality, intubation
AlptekİnoĞlu Mendİl [46] 2021	100	Mixed AHRF (pneumonia 74%)	HFNO (*n* = 51)	SOT(*n*=49)	262	Mortality, intubation
Awadallah [47] 2021	52	ARDS	NIV (*n* = 26)	IMV (*n* = 26)	95	Mortality
Grieco [48] 2021	109	AHRF in COVID-19 patients	NIV (*n* = 54)	HFNO (*n* = 55)	102	Mortality, intubation

*AHRF* acute hypoxemic respiratory failure, *ALI* acute lung injury, *ARDS* acute respiratory distress syndrome, *ARF* acute respiratory failure, *CAP* community-acquired pneumonia, *COPD* chronic obstructive pulmonary disease, *CPE* cardiopulmonary edema, *HFNO* high-flow nasal oxygen, *IMV* invasive mechanical ventilation, *NA* not available, *NIV* noninvasive ventilation, *P/F* ratio ratio of arterial oxygen partial pressure to fractional inspired oxygen, *SOT* standard oxygen therapy

### Risk of bias within studies

Supplementary e-Table 2 shows the data for the risk of bias; 19 studies are judged to have some biased concerns.

### Network meta-analysis

We performed network meta-analyses for mortality and intubation, but not for ventilator-free days and adverse events due to few included studies. The results of pairwise comparisons are shown in Supplementary e-Figures 1 (for mortality), 2 (for intubation), and 3 (for ventilator-associated lung injury). Publication bias was not detected considering the results of the funnel plots (Supplementary e-Fig. 4).

#### Risk of short-term mortality

**Fig. 3:**
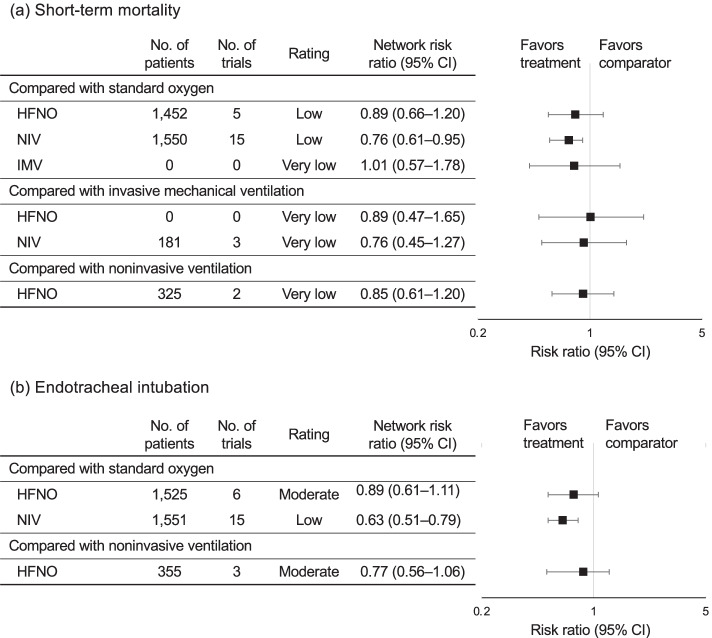
Forest plots for the association of noninvasive oxygenation strategies with study outcomes. **a** For the primary outcome, short-term mortality. **b** For the secondary outcome, endotracheal intubation, all outcomes were reported as network risk ratios and absolute risk differences with 95% confidence intervals (CIs). Risk ratios were estimated for the comparisons of HFNO vs. SOT, NIV vs. SOT, IMV vs. SOT, HFNO vs. IMV, NIV vs. IMV, and HFNO vs. NIV. Abbreviations: CI (confidence interval), HFNO (high-flow nasal oxygen), IMV (invasive mechanical ventilation), NIV (noninvasive ventilation), RR (risk ratio), and SOT (standard oxygen therapy)

In the current analysis for mortality (including 3169 patients across 23 studies), compared to SOT, NIV (RR, 0.76; 95% CI, 0.61–0.95; low certainty) reduced mortality (Fig. 3a). However, IMV (RR, 1.01; 95% CI, 0.57–1.78; very low certainty) and HFNO (RR, 0.89; 95% CI, 0.66–1.20; low certainty) did not reduce mortality. Compared to IMV, HFNO (RR, 0.89; 95% CI, 0.47–1.65; very low certainty) and NIV (RR, 0.76; 95% CI, 0.45–1.27; very low certainty) showed no decrease in mortality risk. The confidence assessment in the RR of each comparison is shown in Table 2.

**Table 2:** Confidence assessment in the risk ratio of each comparison and outcome

	Risk of bias across studies	Imprecision	Heterogeneity	Indirectness	Publication bias	Incoherence	Confidence in relative risk of the event
***Short-term mortality***							
HFNO vs SOT	Undetected	Major concerns ^(a)^ 95% CI (0.66–1.20)	No concerns 95% PI (0.47–1.70)	Low	Not suggested	Some concerns *P*=0.07*	Low
NIV vs SOT	Undetected	No concern 95% CI (0.61–0.95)	Major concerns ^(b)^ 95% PI (0.42–1.39)	Low	Not suggested	No concerns *P*=0.05*	Low
IMV vs SOT	Undetected	Major concerns ^(a)^ 95% CI (0.57–1.78)	No concerns 95% PI (0.45–2.29)	Low	Not suggested	Major concerns^(c)^ *P*= 0.01**	Very low
HFNO vs IMV	Undetected	Major concerns ^(a)^ 95% CI (0.47–1.65)	No concerns 95% PI (0.37–2.10)	Low	Not suggested	Major concerns^(c)^ *P*=0.01**	Very low
NIV vs IMV	Undetected	Major concerns ^(a)^ 95% CI (0.45–1.27)	No concerns 95% PI (0.34–1.65)	Low	Not suggested	Major concern^(c)^ *P*= 0.01**	Very low
NIV vs HFNO	Undetected	Major concerns ^(a)^ 95% CI (0.61–1.20)	No concern 95% PI (0.44–1.67)	Low	Not suggested	Major concern^(c)^ *P*=0.01*	Very low
***Endotracheal intubation***							
HFNO vs SOT	Undetected	Some concerns 95% CI (0.61–1.11)	Some concerns 95% PI (0.41–1.64)	Low	Not suggested	No concern *P*= 0.50*	Moderate
NIV vs SOT	Undetected	No concern 95% CI (0.51–0.79)	Major concerns ^(b)^ 95% PI (0.33–1.22)	Low	Not suggested	No concern *P*= 0.85*	Low
NIV vs HFNO	Undetected	Some concerns 95% CI (0.56–1.06)	Some concerns 95% PI (0.38–1.56)	Low	Not suggested	No concern *P*=0.24*	Moderate

*CI* confidence interval, *SOT* standard oxygenation therapy, *HFNO* high-flow nasal oxygen, *IMV* invasive mechanical ventilation, *NIV* noninvasive ventilation, *PI* prediction interval

^*^We used a side-splitting approach as a local method

^**^We used the design-by-treatment model as a global method

^a^Confidence interval extends into clinically important effects in both directions

^b^*Prediction interval extends into clinically important effects in* both*directions*

^c^*P* value of inconsistency was <0.05

**Fig. 4:**
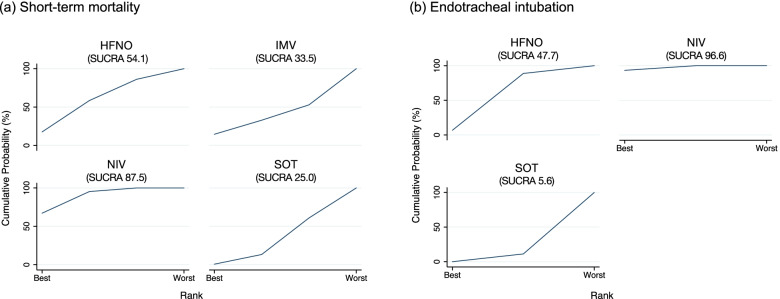
Surface under the cumulative ranking of each noninvasive oxygen strategy for mortality and intubation: **a** short-term mortality, **b** endotracheal intubation. Abbreviations: HFNO (high-flow nasal oxygen), IMV (invasive mechanical ventilation), NIV (noninvasive ventilation), SOT (standard oxygen therapy), and SUCRA (surface under the cumulative ranking)

The ranking analysis revealed that the hierarchy for efficacy in reducing mortality was NIV (SUCRA 87.5), followed by HFNO (SUCRA 54.1), IMV (SUCRA 33.5), and finally, SOT (SUCRA 25.0) (Fig. 4a). As per the results of the current NMA for short-term mortality, we demonstrate the SoF table (Table 3).

**Table 3:**
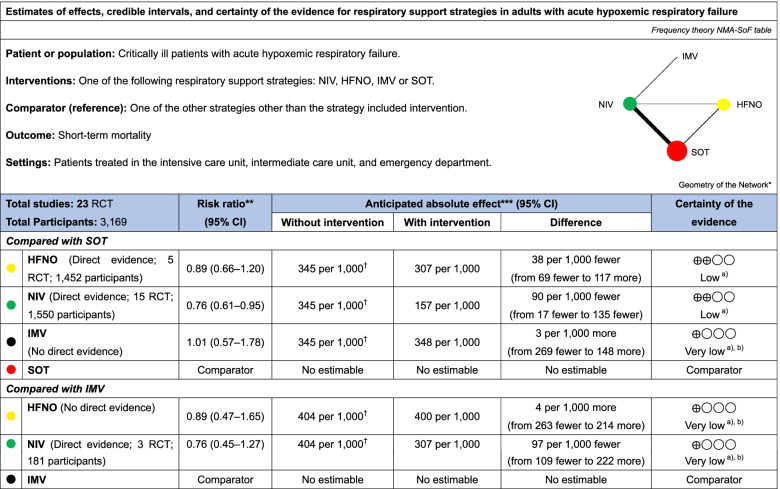
Summary of findings of the network meta-analysis for short-term mortality

*CI* confidence interval, *SOT* standard oxygenation therapy, *HFNO* high-flow nasal oxygen, *IMV* invasive mechanical ventilation, *NIV* noninvasive ventilation, *NMA* network meta-analysis, *PI* prediction interval, *RCT* randomized controlled trial, SoF summary of findings

NMA-SoF table definitions

* Solid lines represent direct comparisons

** Network Metanalysis [54] estimates are reported as risk ratio. *CI* confidence interval

*** Anticipated absolute effect. Anticipated absolute effect compares two risks by calculating the difference between the risk of the intervention group with the risk of the control group

† Information is reported from studies included in the network metanalysis for the comparison displays

**Certainty in the evidence**

**High quality:** We are very confident that the true effect lies close to that of the estimate of the effect

**Moderate quality:** We are moderately confident in the effect estimate: The true effect is likely to be close to the estimate of the effect, but there is a possibility that it is substantially different

**Low quality:** Our confidence in the effect estimate is limited: The true effect may be substantially different from the estimate of the effect

**Very low quality:** We have very little confidence in the effect estimate: The true effect is likely to be substantially different from the estimate of effect

Explanatory Footnotes

a) Serious imprecision

b) Serious incoherence

#### Risk of endotracheal intubation

Twenty-two studies (3118 patients) were included in the analysis for intubation. In comparison with SOT, HFNO (RR, 0.89; 95% CI, 0.61–1.11; moderate certainty) was not associated with a statistically significant lower risk of endotracheal intubation. NIV (RR, 0.63; 95% CI, 0.51–0.79; low certainty) was associated with statistically significant lower risks of endotracheal intubation (Fig. 3b), while no significant difference was observed between NIV and HFNO use in terms of the risk of intubation (RR, 0.77; 95% CI, 0.56–1.06; moderate certainty). The confidence assessment in the RR of each comparison is shown in Table 2.

The ranking analysis revealed that the hierarchy for efficacy in reducing intubation was NIV (SUCRA 96.6), followed by HFNO (SUCRA 47.7), and ultimately SOT (SUCRA 5.6) (Fig. 4b).

#### Other outcomes

For ventilator-free days, an NMA was not conducted because only one trial reported this outcome [12]. Results of the pairwise meta-analysis demonstrated that the incidence of ventilator-associated lung injury was not different between NIV and SOT (RR, 1.38; 95% CI, 0.22–8.54, Supplementary e-Fig. 3). We did not perform a meta-analysis for adverse events, since we did not find any adverse events that were consistently increasing in noninvasive respiratory strategies.

### Sensitivity analysis

As per the results of the sensitivity analyses compared with SOT, the tendency to reduce the risk of mortality was observed when excluded studies involved patients with mild hypoxemia, although not significant (Supplementary e-Table 3 )[12, 25, 26, 29–32, 34, 36, 41, 42, 45, 47, 48]. When studies involving patients with hypercapnia were excluded [12, 26–28, 30, 31, 34–36, 38, 42, 44, 46–48], NIV decreased the risk of mortality, when compared with SOT (Supplementary e-Table 4). For intubation, NIV demonstrated the efficacy in these pre-planned analyses, in contrast, HFNO and IMV were effective in neither analysis. In a post hoc sensitivity analysis excluding studies that reported long-term mortality outcomes (60 days [48], 2 months [29], 90 days [12, 25, 43], and 100 days [37]), NIV decreased the risk of mortality compared with SOT (Supplementary e-Table 5).

## Discussion

In the current NMA of trials among adults with AHRF, in comparison with SOT, NIV was associated with a lower risk of mortality and intubation. Ranking analyses showed that NIV was the best strategy for reducing both outcomes. However, as compared to IMV, NIV and HFNO did not decrease mortality. The results of the sensitivity analyses were similar to those of the main analysis, and the efficacy of NIV was similar in almost all the sensitivity analyses. This NMA is the fourth study to evaluate the effectiveness of NIV and HFNO in patients with ARF. The novelty of our NMA is that we compared noninvasive oxygenation strategies with SOT and IMV.

### Comparison with SOT

Our results were similar to those of previous systematic reviews using NMA [49] but different from those of other NMAs [2, 50]. These differences could be attributed to differences in the study inclusion criteria and, consequently, the studies included in the previous NMAs [2, 49, 50].

Ferreyro et al. [49] reported an NMA describing the effects of noninvasive oxygenation strategies. They showed that treatment with NIV was associated with a lower risk of mortality and intubation, and HFNO decreased the risk of intubation compared to SOT. Their NMA included patients with postoperative respiratory failure or chest trauma. These patients showed various causes of respiratory failure, including atelectasis due to poor pain control, chest wall and lung injury, and pleural effusion. In contrast, we excluded some trials to assess the efficacy of noninvasive oxygenation strategies in patients with AHRF.

Yasuda et al. [50] reported an NMA evaluating the effects of noninvasive oxygenation strategies, and they concluded that NIV and HFNO were associated with a lower risk of endotracheal intubation; however, they observed no significant differences in short-term mortality. Although their study was similar to our study for assessing the efficacy of noninvasive respiratory strategies in patients with de novo AHRF, Yasuda et al. [50] included patients who not only had de novo AHRF but also cardiogenic pulmonary edema, which is an established indication for NIV. Since there was insufficient number of RCTs comparing noninvasive respiratory strategies in only patients with de novo AHRF, we excluded studies in which >50% of patients had acute respiratory failure caused by cardiogenic pulmonary edema. Although pulmonary edema is one of the major causes of AHRF, determining whether it is due to increased hydrostatic pressure or increased permeability is challenging in clinical practice; we included a certain number of patients with heart failure. Thus, our inclusion criteria may be more acceptable in clinical practice.

In the NMA conducted by Zayed et al. [2], RCTs that exclusively enrolled subjects with COPD and cardiogenic pulmonary edema were excluded. Their study seemed to have a higher percentage of de novo AHRF cases. Their NMA demonstrated that NIV was associated with a significant reduction in intubation rates but not mortality as compared with SOT. Furthermore, HFNO was not effective for both outcomes, similar to our results. Considering the results from previous NMAs, our findings imply that the effects of noninvasive respiratory strategies were not robust because the point estimates and confidence intervals varied with differences in the inclusion criteria.

### Comparison with IMV

Another crucial difference between our NMA and the previous NMAs is the comparison of noninvasive respiratory strategies with not only SOT but also IMV. For a healthy lung, spontaneous breathing is associated with improved oxygenation through alveolar recruitment, whereas inspiratory effort in an impaired lung is known to cause patient self-inflicted lung injury (P-SILI) [51]. Since maintaining the tidal volume and inspiratory effort within the appropriate ranges is often difficult during NIV, we should be careful to P-SILI [52]. The post hoc analysis of the LUNG SAFE Trial [6] has reported that severer hypoxemia was a risk for NIV failure among patients with ARDS. In our trial, NIV did not reduce mortality as compared with IMV which was not considered to be lung-protective ventilation. Because excessive tidal volume was reported to be associated with NIV failure in patients with AHRF [53], we should not hesitate to initiate lung protective ventilation via endotracheal intubation if patients are at risk for P-SLI.

### Comparison of noninvasive respiratory support strategies

An RCT evaluating NIV, HFNO, and SOT among patients with de novo AHRF demonstrated that HFNO reduced short-term mortality, although intubation rates were not different [12]. Based on these results, HFNO is preferred for patients with de novo AHRF. However, similar to the results of previous NMAs, our NMA did not show significant differences in the comparison of noninvasive respiratory support strategies [2, 49, 50]. Assigning superiority to these noninvasive devices is also difficult, considering the physiological effects as NIV can provide high positive end-expiratory pressures but may increase dead space ventilation. In an RCT comparing noninvasive respiratory support strategies with SOT [54], NIV use reduced the incidence of intubation, but not mortality, possibly because the prolonged time to intubation weakened the positive effects of avoiding intubation. Both noninvasive respiratory support strategies may contribute to delayed intubation and worsened lung injury [55]. It is necessary to assess these strategies with standardized intubation strategies.

### Limitations

Our current network meta-analysis had several limitations. First, we used indirect comparisons to evaluate the effects of HFNO versus those of IMV, and none of the studies included in the current meta-analysis directly compared IMV and HFNO. This does not reflect the strength of the NMA, which is a property of narrowing the confidence intervals. Second, the mean P/F ratio in the two studies involving comparisons with IMV was lower than that in the studies involving comparisons with SOT. These differences in treatment effects may affect intransitivity and incoherence in a network meta-analysis. Third, a potential source of heterogeneity was the different follow-up times for all-cause mortality in the included studies. In the sensitivity analyses that focused on the 30-day mortality, NIV was associated with lower mortality, similar to the main analysis.

## Conclusions

The current NMA of trials involving adult patients with AHRF showed that in comparison with SOT, only NIV reduced the risk of death, while HFNO and IMV did not. Further investigation, especially a comparison of NIV with HFNO, is warranted.

## Supplementary Information

**Additional file 1: Supplementary e-Table 1**. Search strategy. **Supplementary e-Table 2**. Risk of bias summary. **Supplementary e-Table 3**. Sensitivity analysis excluding studies with P/F ratio ≥ 200. **Supplementary e-Table 4**. Sensitivity analysis excluding studies with Type II respiratory failure. **Supplementary e-Table 5**. Sensitivity analysis for mortality excluding studies that reported long-term mortality. **Supplementary e-Fig. 1**. Forest plots for the pairwise comparison of short-term mortality. **Supplementary e-Fig. 2**. Forest plots for the pairwise comparison of endotracheal intubation. **Supplementary e-Fig. 3**. Forest plots for the pairwise comparison of ventilator associated lung injury. **Supplementary e-Fig. 4**. Funnel plots for each outcome.

## Data Availability

The datasets generated during the current study are available from the corresponding author upon reasonable request.

## Abbreviations

AHRF: Acute hypoxemic respiratory failure
ARDS: Acute respiratory distress syndrome
CENTRAL: Cochrane Central Register of Controlled Trials
CI: Confidence interval
CINeMA: Confidence in Network Meta-Analysis
COPD: Chronic obstructive pulmonary disease
COVID-19: Coronavirus disease 2019
GRADE: Grades of Recommendation, Assessment, Development and Evaluation Working Group
HFNO: High-flow nasal oxygen
ICU: Intensive care unit
IMV: Invasive mechanical ventilation
NIV: Noninvasive ventilation
NMA: Network meta-analysis
PRISMA: Preferred Reporting Items for Systematic Reviews and Meta-Analyses
P-SILI: Patient self-inflicted lung injury
RCT: Randomized controlled trial
RevMan: Review manager
RR: Risk ratio
SOT: Standard oxygen therapy
SUCRA: Surface under the cumulative ranking curve
