# Supplementary material for: Respiratory support strategy in adults with acute hypoxemic respiratory failure: a systematic review and network meta-analysis
Source: JA Clin Rep. 2022 May 6;8:34. doi: 10.1186/s40981-022-00525-4 (PMC9072760; doi:10.1186/s40981-022-00525-4)

Respiratory support strategy in adults with acute hypoxemic respiratory failure: A systematic review and network meta-analysis

Table of Contents

Tables

Supplementary e-Table 1 Search strategy

……………………. 2–5

Supplementary e-Table 2 Risk of bias summary

……………………. 6–8

Supplementary e-Table 3 Sensitivity analysis excluding studies with P/F ratio ≥ 200

..……………………. 9

Supplementary e-Table 4 Sensitivity analysis excluding studies with Type II respiratory failure

..……………………. 10

Supplementary e-Table 5 Sensitivity analysis for mortality excluding studies that reported long-term mortality

..……………………. 11

Figures

Supplementary e-Fig. 1 Forest plots for the pairwise comparison of short-term mortality

..……………………. 12

Supplementary e-Fig. 2 Forest plots for the pairwise comparison of endotracheal intubation

..……………………. 13

Supplementary e-Fig. 3 Forest plots for the pairwise comparison of ventilator associated lung injury

..……………………. 14

Supplementary e-Fig. 4 Funnel plots for each outcome

..……………………. 15

Supplementary e-Table 1 Search strategy a) PubMed search strategy (Performed on May 30, 2021)

#1	"Hypoxia"[mh] OR hypox*[tiab] OR "Respiratory Insufficiency"[mh] OR respiratory depression*[tiab] OR respiratory failure*[tiab] OR ventilatory depression*[tiab] OR respiratory insufficienc*[tiab] OR "Dyspnea"[mh] OR dyspnea*[tiab] OR "shortness of breath"[tiab] OR "Respiratory Distress Syndrome, Adult"[mh] OR acute respiratory distress[tiab] OR adult respiratory distress[tiab] OR respiratory distress syndrome*[tiab] OR RDS[tiab] OR ARDS[tiab] OR "Acute Lung Injury"[mh] OR acute lung injur*[tiab] OR ALI[tiab]
#2	"Respiratory Distress Syndrome, Newborn"[mh]
#3	#1 or #2
#4	"Noninvasive Ventilation"[mh] OR noninvasive ventilation*[tiab] OR non invasive ventilation*[tiab] OR NIV[tiab] OR NPPV[tiab] OR NIPPV[tiab] OR noninvasive positive pressure ventilation*[tiab] OR noninvasive mechanical ventilation*[tiab] OR noninvasive pressure support ventilation*[tiab] OR "Continuous Positive Airway Pressure"[mh] OR continuous positive airway pressure*[tiab] OR bilevel positive airway pressure*[tiab] OR biphasic positive airway pressure*[tiab] OR BIPAP[tiab]
#5	"Oxygen Inhalation Therapy"[mh] OR HFNC[tiab] OR HHFNC[tiab] OR HHHFNC[tiab] OR HFNO[tiab] OR HFNT[tiab] OR HFNOT[tiab] OR HFO[tiab] OR HFOT[tiab] OR NHF[tiab] OR NHFC[tiab] OR NHFT[tiab] OR NHFO[tiab] OR NHFOT[tiab] OR high flow therap*[tiab] OR high flow oxygen[tiab] OR nasal high flow[tiab]
#6	#4 or #5
#7	("Randomized Controlled Trial"[pt] OR "Controlled Clinical Trial"[pt] OR "Clinical Trials as Topic"[mh] OR randomized[tiab] OR placebo[tiab] OR randomly[tiab] OR trial[tiab] OR groups[tiab]) NOT (Animals [mh] NOT Humans [mh])
#8	#3 and #6 and #7

b) CENTRAL search strategy (Performed on May 30, 2021)

ID	Search
#1	[mh Hypoxia] OR hypox*:ti,ab OR [mh "Respiratory Insufficiency"] OR "respiratory depression":ti,ab OR "respiratory failure":ti,ab OR "ventilatory depression":ti,ab OR "respiratory insufficiency":ti,ab OR [mh Dyspnea] OR dyspnea:ti,ab OR "shortness of breath":ti,ab OR [mh "Respiratory Distress Syndrome, Adult"] OR "acute respiratory distress":ti,ab OR "adult respiratory distress":ti,ab OR "respiratory distress syndrome":ti,ab OR RDS:ti,ab OR ARDS:ti,ab OR [mh "Acute Lung Injury"] OR "acute lung injury":ti,ab OR ALI:ti,ab
#2	[mh "Respiratory Distress Syndrome, Newborn"]
#3	#1 OR #2
#4	[mh "Noninvasive Ventilation"] OR "noninvasive ventilation":ti,ab OR "non invasive ventilation":ti,ab OR NIV:ti,ab OR NPPV:ti,ab OR NIPPV:ti,ab OR "noninvasive positive pressure ventilation":ti,ab OR "noninvasive mechanical ventilation":ti,ab OR "noninvasive pressure support ventilation":ti,ab OR [mh "Continuous Positive Airway Pressure"] OR "continuous positive airway pressure":ti,ab OR "bilevel positive airway pressure":ti,ab OR "biphasic positive airway pressure":ti,ab OR BIPAP:ti,ab
#5	[mh "Oxygen Inhalation Therapy"] OR HFNC:ti,ab OR HHFNC:ti,ab OR HHHFNC:ti,ab OR HFNO:ti,ab OR HFNT:ti,ab OR HFNOT:ti,ab OR HFO:ti,ab OR HFOT:ti,ab OR NHF:ti,ab OR NHFC:ti,ab OR NHFT:ti,ab OR NHFO:ti,ab OR NHFOT:ti,ab OR "high flow therapy":ti,ab OR "high flow oxygen":ti,ab OR "nasal high flow":ti,ab
#6	#4 OR #5
#7	#3 AND #6
#8	[mh animals] NOT [mh humans]
#9	#7 NOT #8

c) Embase search strategy (Performed on May 30, 2021)

Set#	Searched for
S1	(EMB.EXACT("hypoxia") OR (EMB.EXACT("paroxysmal dyspnea") OR EMB.EXACT("dyspnea")) OR (EMB.EXACT.EXPLODE("respiratory failure")))
S2	(EMB.EXACT.EXPLODE("neonatal respiratory distress syndrome") OR EMB.EXACT.EXPLODE("acute lung injury") OR EMB.EXACT.EXPLODE("adult respiratory distress syndrome"))
S3	(TI,AB(hypoxi* OR (respiratory p/0 (depression* OR failure* OR insufficienc*)) OR (ventilatory p/0 depression*) OR dyspnea* OR "shortness of breath" OR "acute respiratory distress" OR "adult respiratory distress" OR "respiratory distress syndrome" OR RDS OR ARDS OR (acute p/0 lung p/0 injur*) OR ALI))
S4	(S1 or S2 or S3)
S5	(EMB.EXACT("noninvasive ventilation"))
S6	(TI,AB("noninvasive ventilation" OR "non invasive ventilation" OR NIV OR NPPV OR NIPPV OR "noninvasive positive pressure ventilation" OR "noninvasive mechanical ventilation" OR "noninvasive pressure support ventilation" OR "continuous positive airway pressure" OR "bilevel positive airway pressure" OR "biphasic positive airway pressure" OR BIPAP))
S7	(EMB.EXACT("home oxygen therapy") OR EMB.EXACT("hyperbaric oxygen therapy") OR EMB.EXACT("oxygen therapy"))
S8	(TI,AB(HFNC OR HHFNC OR HHHFNC OR HFNO OR HFNT OR HFNOT OR HFO OR HFOT OR NHF OR NHFC OR NHFT OR NHFO OR NHFOT OR ("high flow" p/0 (therap* OR oxygen)) OR "nasal high flow"))
S9	(S5 or S6 or S7 or S8)
S10	((EMB.EXACT("controlled clinical trial") OR EMB.EXACT.EXPLODE("clinical trial (topic)") OR EMB.EXACT("randomized controlled trial")) OR (TI,AB(randomized) OR TI,AB(randomly) OR TI(trial) OR TI,AB(groups)) NOT (ANIMAL(YES) NOT HUMAN(YES)))
S11	(S4 and S9 and S10)
S12	(S11 AND PD(>=2010))
S13	(S11 AND PD(<2010))

d) Ichushi search strategy (Performed on May 30, 2021)

Search	Query
#1	酸素欠乏/TH or 酸素欠乏/TA or anoxia/TA or Hypoxia/TA
#2	呼吸窮迫症候群-急性/TH or 急性呼吸窮迫症候群/TA or ARDS/TA
#3	急性肺損傷/TH or 急性肺損傷/TA or 急性肺障害/TA or 急性肺傷害/TA or ALI/TA
#4	呼吸不全/TH or 呼吸不全/TA
#5	呼吸困難/TH or 呼吸困難/TA
#6	呼吸窮迫症候群-新生児/TH or 新生児呼吸窮迫症候群/TA
#7	#1 or #2 or #3 or #4 or #5 or #6
#8	非侵襲的補助換気/TH or 非侵襲的補助換気/TA or NPPV/TA or NIPPV/TA
#9	持続気道陽圧/TH or 持続気道陽圧/TA or CPAP/TA
#10	非侵襲的陽圧呼吸/TH or 非侵襲的陽圧呼吸/TA or BIPAP/TA
#11	酸素吸入療法/TH or 酸素吸入/TA
#12	酸素療法/TA or ハイフロー/TA or HFNC/TA or NHF/TA or HFO/TA
#13	#8 or #9 or #10 or #11 or #12
#14	#7 and #13
#15	(#14) and (PT=会議録除く)
#16	ランダム化比較試験/TH or ランダム化/AL or 無作為化/AL
#17	比較試験/AL
#18	臨床試験/TH or 臨床試験/AL
#19	プラセボ/TH or プラセボ/AL
#20	対照/AL
#21	コントロール/AL
#22	臨床研究・疫学研究/TH or 臨床研究/AL
#23	#16 or #17 or #18 or #19 or #20 or #21 or #22
#24	#15 and #23

Supplementary e-Table 2 Risk of bias summary

(a) Short-term mortality

Source	Bias arising from the randomization process	Bias due to deviations from intended interventions	Bias due to missing outcome data	Bias in measurement of the outcome	Bias in selection of the reported result	Overall risk of bias
Wysock 1995	Low	Some concerns	Low	Low	Low	Some concerns
Antolnelli 1998	Some concerns	Some concerns	Low	Low	Low	High
Confalonieri 1999	Low	Some concerns	Low	Low	Low	Some concerns
Antonelli 2000	Low	Some concerns	Low	Low	Low	Some concerns
Delcaux 2000	Low	Some concerns	Some concerns	Low	Low	High
Martin 2000	Some concerns	Some concerns	Low	Low	Low	Hugh
Hilbert 2001	Low	Some concerns	Low	Low	Low	Some concerns
Ferrer 2003	Low	Some concerns	Low	Low	Low	Some concerns
Cosentini 2010	Low	Some concerns	Low	Low	Low	Some concerns
Squadrone 2010	Low	Some concerns	Low	Low	Low	Some concerns
Wermke 2012	Low	Some concerns	Some concerns	Low	Low	High
Zhan 2012	Low	Some concerns	Low	Low	Low	Some concerns
Brambilila 2014	Low	Some concerns	Low	Low	Low	Some concerns
Frat 2015	Low	Some concerns	Low	Low	Low	Some concerns
Lemiale2015_JAMA	Low	Some concerns	Low	Low	Low	Some concerns
Jones 2016	Low	Some concerns	Low	Low	Low	Some concerns
Muncharaz 2017	Low	Some concerns	Low	Low	Low	Some concerns
Azoulay 2018	Low	Some concerns	Low	Low	Low	Some concerns
He 2019	Low	Some concerns	Low	Low	Low	Some concerns
Andino 2020	Low	Some concerns	Low	Low	Low	Some concerns

AlptekİnoĞlu Mendİl 2021	Some concerns	Some concerns	Low	Low	Low	High
Awadallah 2021	Some concerns	Some concerns	Low	Low	Low	Some concerns
Groeco 2021	Some concerns	Some concerns	Low	Low	Low	High

(b) Endotracheal intubation

Source	Bias arising from the randomization process	Bias due to deviations from intended interventions	Bias due to missing outcome data	Bias in measurement of the outcome	Bias in selection of the reported result	Overall risk of bias
Wysock 1995	Low	Some concerns	Low	Low	Low	Some concerns
Confalonieri 1999	Low	Some concerns	Low	Low	Low	Some concerns
Antonelli 2000	Low	Some concerns	Low	Low	Low	Some concerns
Delcaux 2000	Low	Some concerns	Some concerns	Low	Low	High
Martin 2000	Some concerns	Some concerns	Low	Low	Low	High
Hilbert 2001	Low	Some concerns	Low	Low	Low	Some concerns
Ferrer 2003	Low	Some concerns	Low	Low	Low	Some concerns
Cosentini 2010	Low	Some concerns	Low	Low	Low	Some concerns
Squadrone 2010	Low	Some concerns	Low	Low	Low	Some concerns
Wermke 2012	Low	Some concerns	Some concerns	Low	Low	High
Zhan 2012	Low	Some concerns	Low	Low	Low	Some concerns
Brambilila 2014	Low	Some concerns	Low	Low	Low	Some concerns
Azevedo 2015	Some concerns	Some concerns	Low	Low	Low	High
Frat 2015	Low	Some concerns	Low	Low	Low	Some concerns
Lamiale 2015_CC	Low	Some concerns	Low	Low	Low	Some concerns
Lemiale2015_JAMA	Low	Some concerns	Low	Low	Low	Some concerns
Jones 2016	Low	Some concerns	Low	Low	Low	Some concerns
Azoulay 2018	Low	Some concerns	Low	Low	Low	Some concerns
He 2019	Low	Some concerns	Low	Low	Low	Some concerns
Andino 2020	Low	Some concerns	Low	Low	Low	Some concerns
AlptekİnoĞlu Mendİl 2021	Low	Some concerns	Low	Low	Low	Some concerns
Groeco 2021	Some concerns	Some concerns	Low	Low	Low	High

Supplementary e-Table 3 Sensitivity analysis excluding studies with P/F ratio ≥ 200

(a) Short-term mortality

Comparison	All studies-Main analysis			Excluding studies with P/F ratio ≥ 200		
	No. of studies	RR (95% CI)	Rating	No. of studies	RR (95% CI)	Rating
HFNO vs SOT	5	0.89 (0.66–1.20)	Low	5	0.65 (0.35–1.20)	Low
NIV vs SOT	15	0.76 (0.61–0.95)	Low	8	0.67 (0.42–1.05)	Low
IMV vs SOT	0	1.01 (0.57–1.78)	Very low	0	1.07 (0.41–2.79)	Low
HFNO vs IMV	0	0.89 (0.78–1.65)	Very low	0	0.61 (0.21–1.77)	Low
NIV vs IMV	3	0.76 (0.45–1.27)	Very low	3	0.62 (0.27–1.45)	Low
NIV vs HFNO	2	0.85 (0.61–1.20)	Very low	2	1.03 (0.53–1.99)	Low

Testing for global incoherence: Overall RCTs, P= 0.01; Excluding studies with P/F ≥ 200, P=0.13.

(b) Endotracheal intubation

Comparison	All patients-Main analysis			Excluding studies with P/F ratio ≥ 200		
	No. of studies	RR (95% CI)	Rating	No. of studies	RR (95% CI)	Rating
HFNO vs SOT	6	0.89 (0.61–1.11)	Moderate	4	0.77 (0.54–1.10)	Low
NIV vs SOT	15	0.63 (0.51–0.79)	Low	8	0.62 (0.48–0.81)	Low
NIV vs HFNO	3	0.77 (0.56–1.06)	Moderate	2	0.80 (0.55–1.17)	Low

Testing for global incoherence: Overall RCTs, P= 0.09; Excluding studies with P/F ≥ 200, P=0.09

Supplementary e-Table 4 Sensitivity analysis excluding studies with Type II respiratory failure

(a) Short-term mortality

Comparison	All studies-Main analysis			Excluding studies with Type II respiratory failure		
	No. of studies	RR (95% CI)	Rating	No. of studies	RR (95% CI)	Rating
HFNO vs SOT	5	0.89 (0.66–1.20)	Low	3	0.73 (0.43–1.25)	Low
NIV vs SOT	15	0.76 (0.61–0.95)	Low	11	0.69 (0.49–0.98)	Low
IMV vs SOT	0	1.01 (0.57–1.78)	Very low	0	0.94 (0.45–1.93)	Very low
HFNO vs IMV	0	0.89 (0.78–1.65)	Very low	0	0.78 (0.45–1.82)	Very low
NIV vs IMV	3	0.76 (0.45–1.27)	Very low	3	0.75 (0.39–1.40)	Very low
NIV vs HFNO	2	0.85 (0.61–1.20)	Very low	2	0.94 (0.54–1.64)	Very low

Testing for global incoherence: Overall RCTs, P= 0.01; Excluding studies with Type II respiratory failure, P=0.17.

(b) Endotracheal intubation

Comparison	All patients-Main analysis			Excluding studies with Type II respiratory failure		
	No. of studies	RR (95% CI)	Rating	No. of studies	RR (95% CI)	Rating
HFNO vs SOT	6	0.89 (0.61–1.11)	Moderate	4	0.81 (0.50–1.32)	Low
NIV vs SOT	15	0.63 (0.51–0.79)	Low	11	0.61 (0.44–0.85)	Low
NIV vs HFNO	3	0.77 (0.56–1.06)	Moderate	2	0.75 (0.46–1.24)	Low

Testing for global incoherence: Overall RCTs, P= 0.30; Excluding studies with Type II respiratory failure, P=0.42

Supplementary e-Table 5 Sensitivity analysis for mortality excluding studies that reported long-term mortality

Comparison	All studies-Main analysis			Excluding studies with long term mortality		
	No. of studies	RR (95% CI)	Rating	No. of studies	RR (95% CI)	Rating
HFNO vs SOT	5	0.89 (0.66–1.20)	Low	2	1.26 (0.69–2.31)	Very low
NIV vs SOT	15	0.76 (0.61–0.95)	Low	12	0.64 (0.49–0.83)	Very low
IMV vs SOT	0	1.01 (0.57–1.78)	Very low	0	0.84 (0.47–1.47)	Very low
HFNO vs IMV	0	0.89 (0.78–1.65)	Very low	0	1.50 (0.65–3.48)	Very low
NIV vs IMV	3	0.76 (0.45–1.27)	Very low	3	0.76 (0.45–1.27)	Very low
NIV vs HFNO	2	0.85 (0.61–1.20)	Very low	0	0.50 (0.54–3.79)	Very low

Testing for global incoherence: Overall RCTs, P= 0.01; Excluding studies with long mortality, P=0.06.

Supplementary e-Fig. 1 Forest plots for the pairwise comparison of short-term mortality

(a) HFNO vs SOT

	HFNO		SOT			Risk Ratio	
Study or Subgroup	Events	Total	Events	Total	Weight	IV, Random, 95% CI	Year
Frat 2015	13	106	22	94	11.4%	0.52 [0.28, 0.98]	2015
Jones 2016	35	165	24	138	17.8%	1.22 [0.76, 1.95]	2016
Azoulay 2018	182	388	187	388	49.5%	0.97 [0.84, 1.13]	2018
Andino 2020	6	24	4	22	4.0%	1.38 [0.45, 4.24]	2020
AlptekinoĞlu Mendİl 2021	23	51	18	49	17.3%	1.23 [0.76, 1.98]	2021
**Total (95% CI)**		**734**		**691**	**100.0%**	**1.00 [0.79, 1.26]**	
Total events	259		255				

Heterogeneity: Tau² = 0.02; Chi² = 5.88, df = 4 (P = 0.21); I² = 32%
Test for overall effect: Z = 0.03 (P = 0.98)

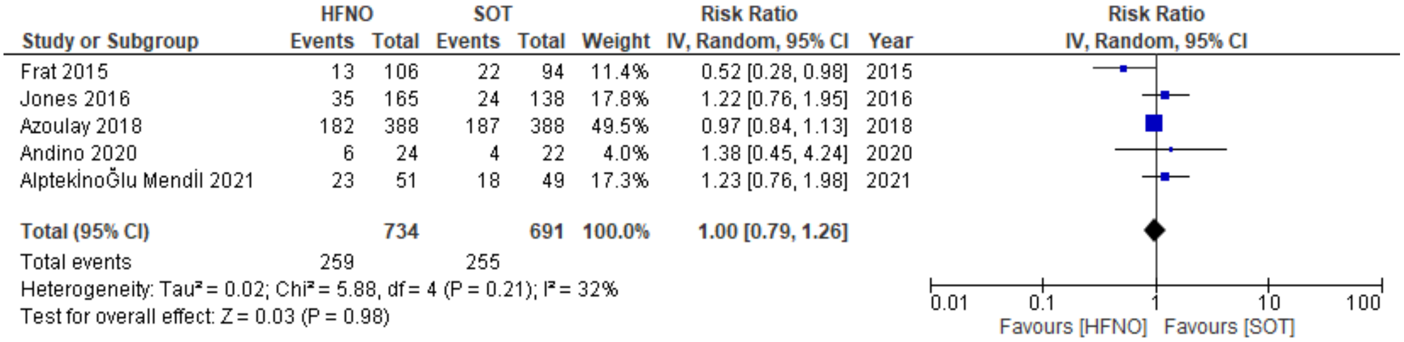

(b) NIV vs SOT

	NIV		SOT			Risk Ratio	
Study or Subgroup	Events	Total	Events	Total	Weight	IV, Random, 95% CI	Year
Wysock 1995	7	21	10	20	5.9%	0.67 [0.32, 1.41]	1995
Confalonieri 1999	7	28	10	28	5.2%	0.70 [0.31, 1.58]	1999
Martin 2000	5	32	10	29	4.1%	0.45 [0.18, 1.17]	2000
Antonelli 2000	7	20	11	20	6.3%	0.64 [0.31, 1.30]	2000
Delcaux 2000	19	62	18	61	9.3%	1.04 [0.61, 1.78]	2000
Hilbert 2001	13	26	21	26	12.0%	0.62 [0.40, 0.95]	2001
Ferrer 2003	9	51	21	54	6.8%	0.45 [0.23, 0.90]	2003
Squadrone 2010	3	20	15	20	3.3%	0.20 [0.07, 0.59]	2010
Cosentini 2010	0	20	0	27		Not estimable	2010
Wermke 2012	26	42	30	44	15.6%	0.91 [0.66, 1.24]	2012
Zhan 2012	1	21	5	19	1.0%	0.18 [0.02, 1.41]	2012
Brambilila 2014	2	40	7	41	1.8%	0.29 [0.06, 1.33]	2014
Frat 2015	31	119	22	94	10.7%	1.11 [0.69, 1.79]	2015
Lemiale (JAMA) 2015	46	191	50	183	14.5%	0.88 [0.62, 1.25]	2015
He 2019	7	102	7	98	3.6%	0.96 [0.35, 2.64]	2019
**Total (95% CI)**		**795**		**764**	**100.0%**	**0.72 [0.59, 0.89]**	
Total events	183		237				

Heterogeneity: Tau² = 0.05; Chi² = 19.87, df = 13 (P = 0.10); I² = 35%
Test for overall effect: Z = 3.05 (P = 0.002)

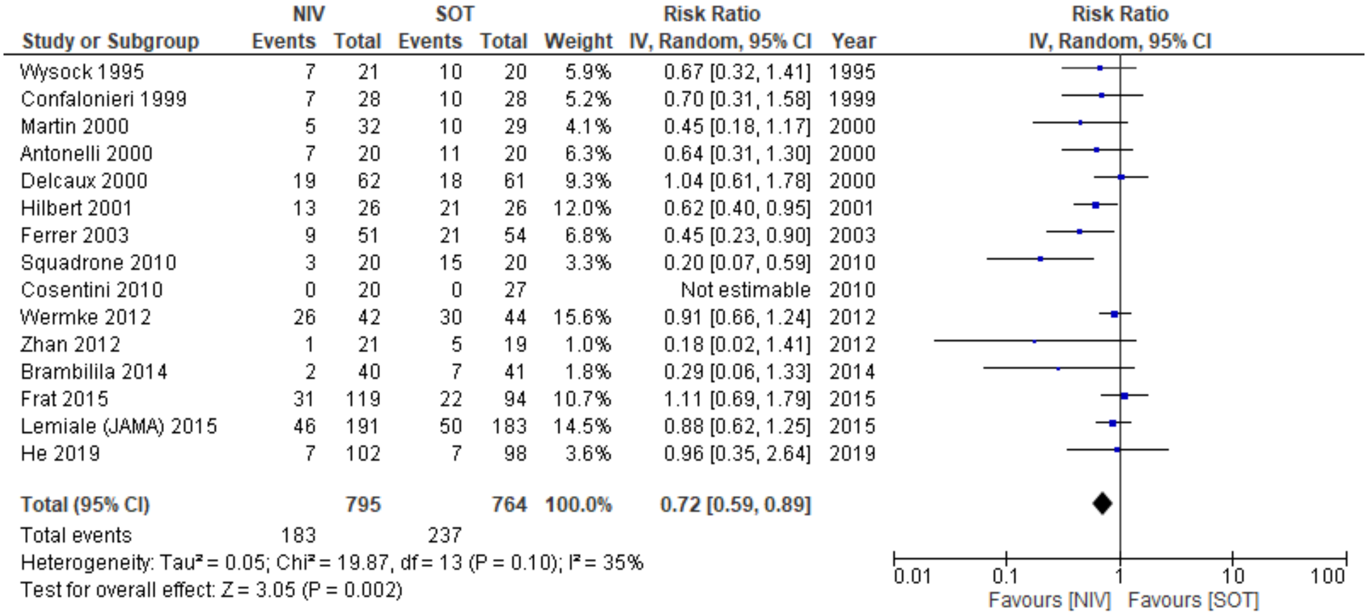

(c) NIV vs IMV

	NIV		IMV			Risk Ratio	
Study or Subgroup	Events	Total	Events	Total	Weight	IV, Random, 95% CI	Year
Antolnelli 1998	10	32	16	32	37.9%	0.63 [0.34, 1.16]	1998
Muncharaz 2017	14	34	9	31	35.9%	1.42 [0.72, 2.80]	2017
Awadallah 2021	4	26	11	26	26.2%	0.36 [0.13, 1.00]	2021
**Total (95% CI)**		**92**		**89**	**100.0%**	**0.73 [0.35, 1.51]**	
Total events	28		36				

Heterogeneity: Tau² = 0.27; Chi² = 5.65, df = 2 (P = 0.06); I² = 65%
Test for overall effect: Z = 0.85 (P = 0.39)

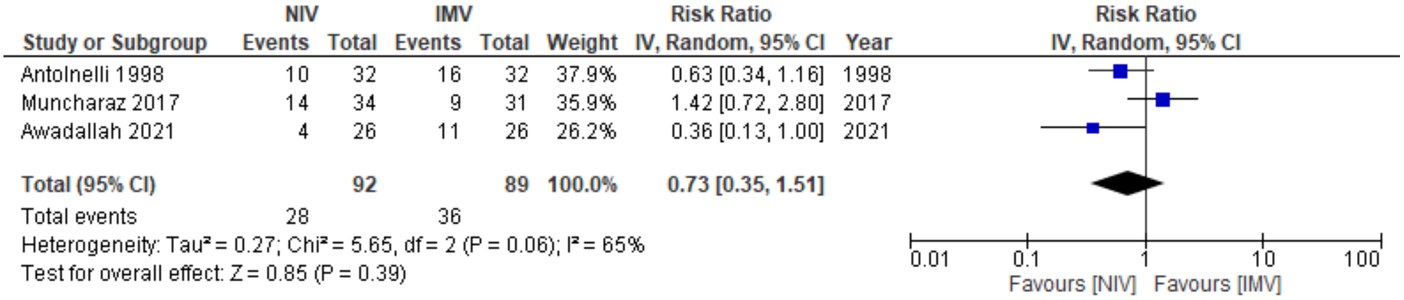

(d) NIV vs HFNO

	NIV		HFNO			Risk Ratio	
Study or Subgroup	Events	Total	Events	Total	Weight	IV, Random, 95% CI	Year
Frat 2015	31	110	13	106	53.1%	2.30 [1.27, 4.15]	2015
Grieco 2021	13	54	12	55	46.9%	1.10 [0.55, 2.20]	2021
**Total (95% CI)**		**164**		**161**	**100.0%**	**1.63 [0.79, 3.34]**	
Total events	44		25				

Heterogeneity: Tau² = 0.16; Chi² = 2.51, df = 1 (P = 0.11); I² = 60%
Test for overall effect: Z = 1.33 (P = 0.18)

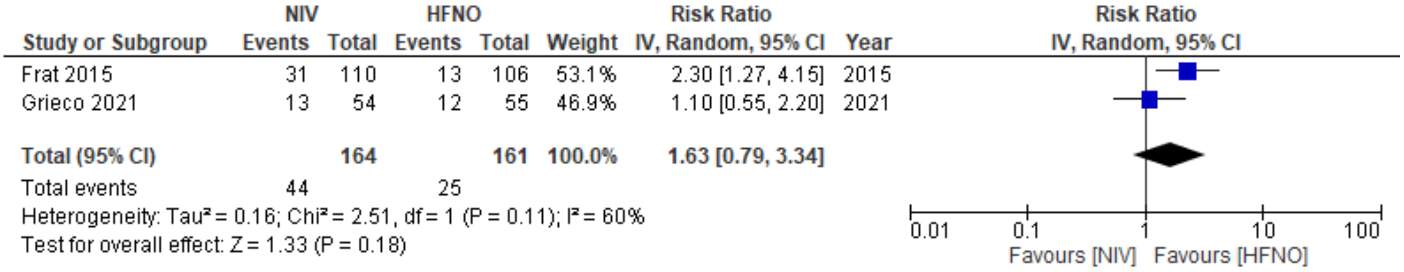

Supplementary e-Fig. 2 Forest plots for the pairwise comparison of endotracheal intubation

(a) HFNO vs SOT

Study or Subgroup	HFNO Events	HFNO Total	SOT Events	SOT Total	Weight	Risk Ratio IV, Random, 95% CI	Year
Frat 2015	40	106	44	94	28.9%	0.81 [0.58, 1.12]	2015
Lamiale (CC) 2015	4	52	2	48	2.3%	1.85 [0.35, 9.63]	2015
Jones 2016	1	165	3	138	1.3%	0.28 [0.03, 2.65]	2016
Azoulay 2018	150	388	170	388	44.2%	0.88 [0.75, 1.04]	2018
Andino 2020	8	24	14	22	12.1%	0.52 [0.27, 1.00]	2020
AlptekİnoĞlu Mendİl 2021	17	51	10	49	11.3%	1.63 [0.83, 3.21]	2021
**Total (95% CI)**		**786**		**739**	**100.0%**	**0.87 [0.67, 1.12]**	
Total events	220		243				

Heterogeneity: Tau² = 0.03; Chi² = 7.71, df = 5 (P = 0.17); I² = 35%
Test for overall effect: Z = 1.09 (P = 0.27)

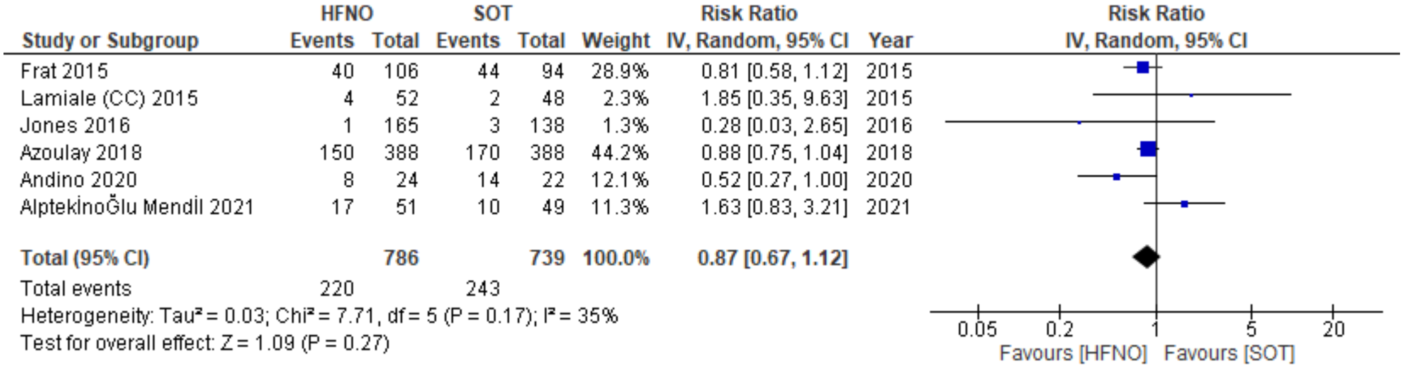

(b) NIV vs SOT

Study or Subgroup	NIV Events	NIV Total	SOT Events	SOT Total	Weight	Risk Ratio IV, Random, 95% CI	Year
Wysock 1995	13	21	14	20	9.8%	0.88 [0.57, 1.38]	1995
Confalonieri 1999	6	28	17	28	6.1%	0.35 [0.16, 0.76]	1999
Antonelli 2000	4	20	14	20	4.9%	0.29 [0.11, 0.72]	2000
Delcaux 2000	21	62	24	61	9.5%	0.86 [0.54, 1.37]	2000
Martin 2000	9	32	17	29	7.5%	0.48 [0.25, 0.90]	2000
Hilbert 2001	12	26	20	26	9.5%	0.60 [0.38, 0.96]	2001
Ferrer 2003	13	51	28	54	8.6%	0.49 [0.29, 0.84]	2003
Cosentini 2010	0	20	0	27		Not estimable	2010
Squadrone 2010	2	20	8	20	2.6%	0.25 [0.06, 1.03]	2010
Wermke 2012	6	43	11	44	5.0%	0.56 [0.23, 1.37]	2012
Zhan 2012	1	21	4	19	1.3%	0.23 [0.03, 1.85]	2012
Brambilila 2014	6	40	26	41	6.1%	0.24 [0.11, 0.51]	2014
Frat 2015	55	110	44	94	11.9%	1.07 [0.80, 1.42]	2015
Lemiale (JAMA) 2015	73	191	82	183	12.5%	0.85 [0.67, 1.09]	2015
He 2019	9	102	7	98	4.7%	1.24 [0.48, 3.19]	2019
**Total (95% CI)**		**787**		**764**	**100.0%**	**0.61 [0.48, 0.79]**	
Total events	230		316				

Heterogeneity: Tau² = 0.12; Chi² = 34.51, df = 13 (P = 0.001); I² = 62%
Test for overall effect: Z = 3.81 (P = 0.0001)

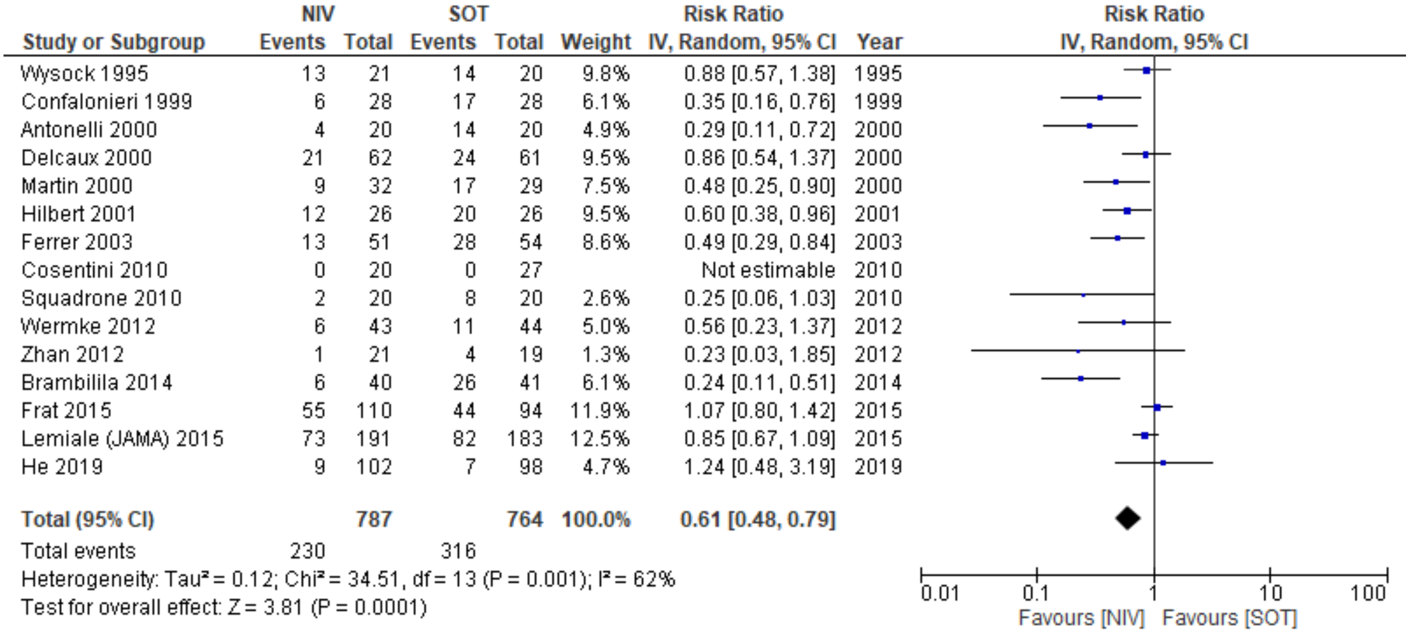

(c) NIV vs HFNO

Study or Subgroup	NIV Events	NIV Total	HFNO Events	HFNO Total	Weight	Risk Ratio IV, Random, 95% CI	Year
Frat 2015	55	110	40	106	38.8%	1.32 [0.97, 1.80]	2015
Azevedo 2015	9	16	9	14	28.9%	0.88 [0.49, 1.57]	2015
Grieco 2021	16	54	28	55	32.4%	0.58 [0.36, 0.95]	2021
**Total (95% CI)**		**180**		**175**	**100.0%**	**0.90 [0.53, 1.52]**	
Total events	80		77				

Heterogeneity: Tau² = 0.16; Chi² = 8.16, df = 2 (P = 0.02); I² = 75%
Test for overall effect: Z = 0.39 (P = 0.70)

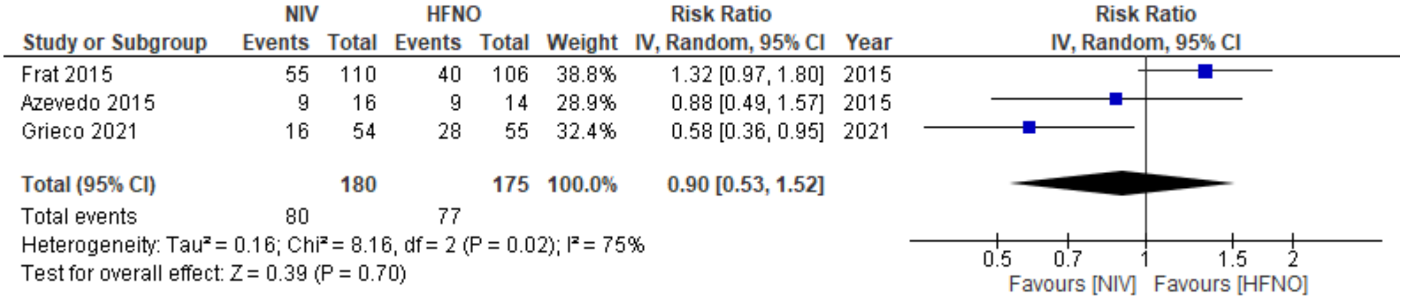

Supplementary e-Fig. 3 Forest plots for the pairwise comparison of ventilator associated lung injury

	NIV		SOT			Risk Ratio	
**Study or Subgroup**	**Events**	**Total**	**Events**	**Total**	**Weight**	**M-H, Random, 95% CI**	**Year**
Confalonieri 1999	1	28	0	28	33.4%	3.00 [0.13, 70.64]	1999
Delcaux 2000	1	62	0	61	32.9%	2.95 [0.12, 71.09]	2000
Zhan 2012	0	21	1	19	33.7%	0.30 [0.01, 7.02]	2012
**Total (95% CI)**		**111**		**108**	**100.0%**	**1.38 [0.22, 8.54]**	
Total events	2		1				

Heterogeneity: Tau² = 0.00; Chi² = 1.35, df = 2 (P = 0.51); I² = 0%
Test for overall effect: Z = 0.34 (P = 0.73)

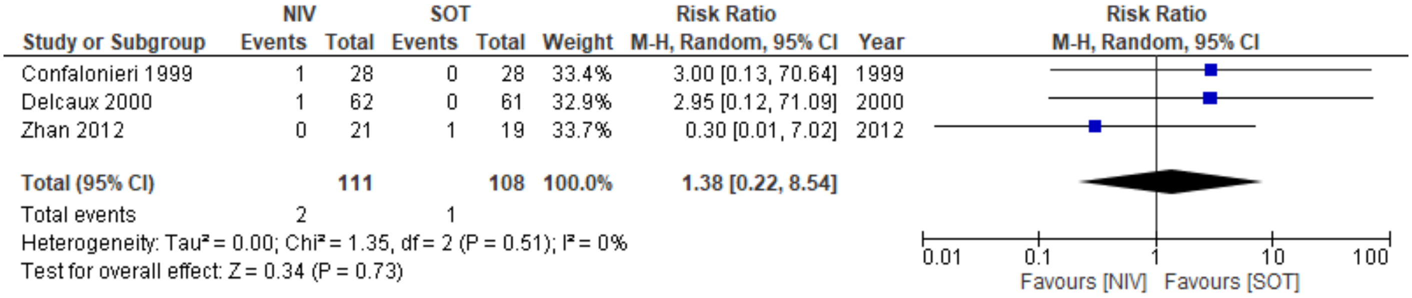

Supplementary e-Fig. 4 Funnel plots for each outcome

(a) Short-term mortality

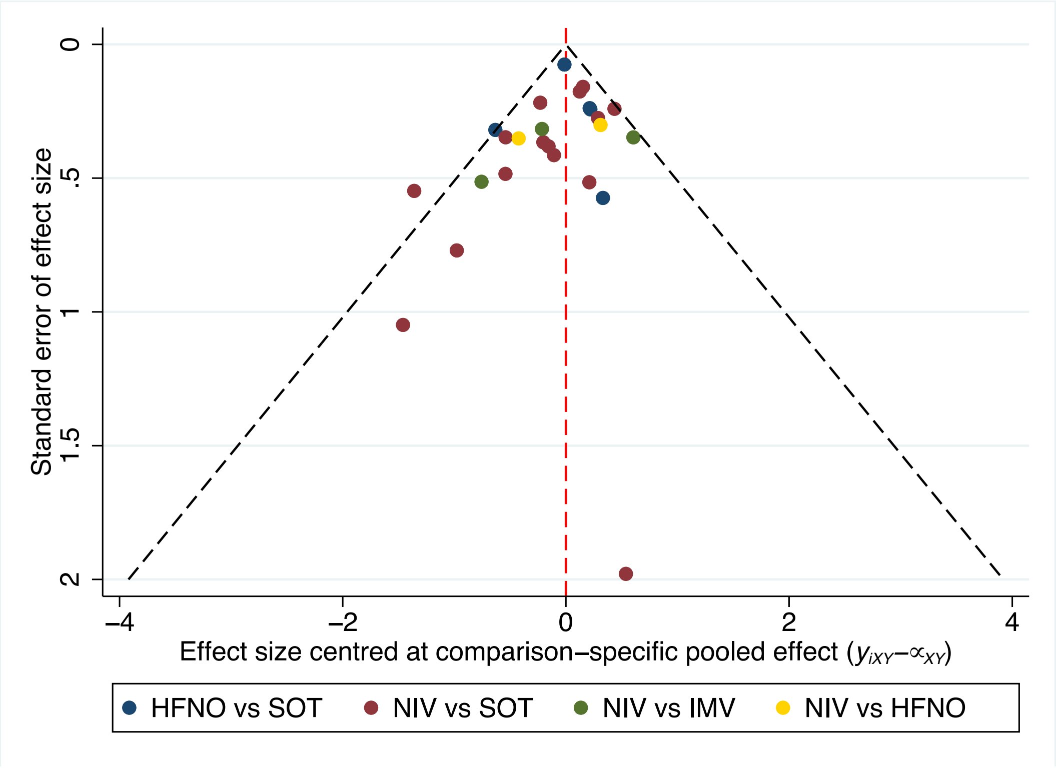

(b) Endotracheal intubation

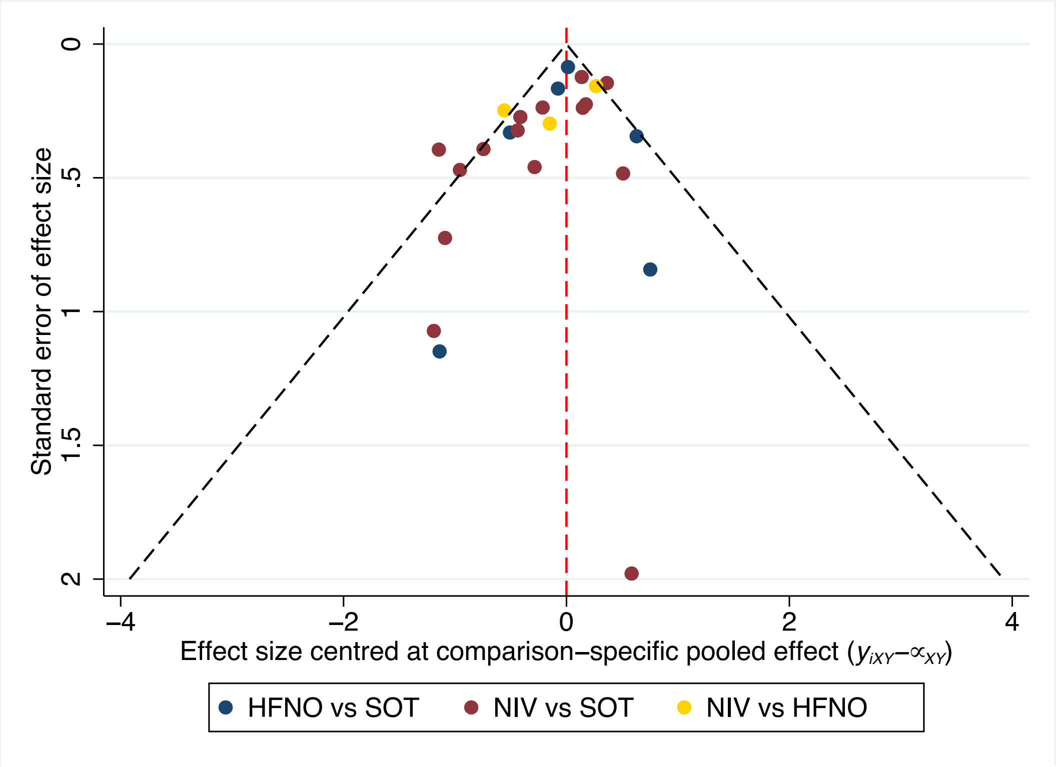

Supplement: Supplementary file 1 — Additional file 1: Supplementary e-Table 1. Search strategy. Supplementary e-Table 2. Risk of bias summary. Supplementary e-Table 3. Sensitivity analysis excluding studies with P/F ratio ≥ 200. Supplementary e-Table 4. Sensitivity analysis excluding studies with Type II respiratory failure. Supplementary e-Table 5. Sensitivity analysis for mortality excluding studies that reported long-term mortality. Supplementary e-Fig. 1. Forest plots for the pairwise comparison of short-term mortality. Supplementary e-Fig. 2. Forest plots for the pairwise comparison of endotracheal intubation. Supplementary e-Fig. 3. Forest plots for the pairwise comparison of ventilator associated lung injury. Supplementary e-Fig. 4. Funnel plots for each outcome. [file 40981_2022_525_MOESM1_ESM.pdf]
